# AC–DC electropenetrography unmasks fine temporal details of feeding behaviors for two tick species on unsedated hosts

**DOI:** 10.1038/s41598-020-80257-6

**Published:** 2021-01-21

**Authors:** Kathryn E. Reif, Elaine A. Backus

**Affiliations:** 1 Department of Diagnostic Medicine/Pathobiology, College of Veterinary Medicine, Kansas State University, Manhattan, KS 66506-5802 USA; 2 USDA Agricultural Research Service, San Joaquin Valley Agricultural Sciences Center, 9611 South Riverbend Ave., Parlier, CA 93648 USA

**Keywords:** Entomology, Biological techniques

## Abstract

Ticks are significant nuisance pests and vectors of pathogens for humans, companion animals, and livestock. Limited information on tick feeding behaviors hampers development and rigorous evaluation of tick and tick-borne pathogen control measures. To address this obstacle, the present study examined the utility of AC–DC electropenetrography (EPG) to monitor feeding behaviors of adult *Dermacentor variabilis* and *Amblyomma americanum* in real-time. EPG recording was performed during early stages of slow-phase tick feeding using an awake calf host. Both tick species exhibited discernable and stereotypical waveforms of low-, medium-, and high-frequencies. Similar waveform families and types were observed for both tick species; however, species-specific waveform structural differences were also observed. Tick waveforms were hierarchically categorized into three families containing seven types. Some waveform types were conserved by both species (e.g., Types 1b, 1c, 2b, 2c) while others were variably performed among species and individually recorded ticks (e.g., Types 1a, 2a, 2d). This study provides a proof-of-principle demonstration of the feasibility for using EPG to monitor, evaluate, and compare tick feeding behaviors, providing a foundation for future studies aimed at correlating specific feeding behaviors with waveforms, and ultimately the influence of control measures and pathogens on tick feeding behaviors.

## Introduction

Ticks are globally and economically significant pests of humans, companion animals, and livestock. Billions of dollars are spent annually trying to control tick populations and manage tick-borne diseases (TBDs) of medical and veterinary concern. In the United States (U.S.), TBDs are the most common vector-borne diseases of people, with 59,349 TBD cases reported to the Centers for Disease Control and Prevention in 2017, an approximate 200% increase in the last two decades^1–4^. The annual healthcare cost of Lyme disease alone is estimated at $712 million to $1.3 billion^1,5^. Because no TBD vaccines are licensed for humans in the U.S., control measures to prevent TBDs center around repelling or killing ticks. For U.S. companion animals, tick-borne pathogen (TBP) seroprevalence rates are also high with 5.64%, 3.23%, and 2.94% of dogs seropositive for Lyme disease, anaplasmosis, and ehrlichiosis agents, respectively, in 2018^6^. In the same year, revenue from the systemically-acting isoxazoline class of ectoparasiticides represented the major share of the $8.65 billion global animal parasiticide market^7^. Finally, ticks are globally the most significant vectors of disease for livestock, with approximately 80% of the world’s cattle populations infected or at risk for TBPs, resulting in an annual economic loss of $19 billion^8,9^. Despite the medical and economic importance of ticks, effective control measures are limited, and concern over the longevity of those currently available highlights the imperative need for development of new mitigation strategies.

A significant knowledge gap exists regarding the details of tick feeding behavior and associated host interactions at and within the feeding lesion because activities such as mouthpart movements, tissue damage, and salivation occur within opaque host tissue, masked from ready observation or investigation. This gap in knowledge presents a significant obstacle to the development and rigorous evaluation of tick and TBP control products. Further complicating the development and evaluation of tick and TBP control strategies is the long duration of time over which ticks feed upon their host, taxing the ability to maintain continuous observation^10^. To facilitate attachment to the host and feeding, as well as to avoid recognition by the host’s immune system, ticks salivate a highly coordinated and wide array of pharmacological compounds to achieve their objective—a successful bloodmeal^10^. Although coordinated salivation processes are generally understood at a coarse time scale, from numerous molecular and biochemical studies^11–16^, the time-scale temporal dynamics of specific tick salivation and ingestion events are largely unknown. Until ‘normal’ tick feeding behaviors and tick-host interactions are delineated, including the temporal sequence of these events, it is nearly impossible to attain a clear understanding of how tick and TBP intervention methods alter tick feeding behavior and tick-host interactions^17^.

Electropenetrography (EPG; not to be confused with ‘electrophysiology’ also sometimes abbreviated EPG) is an electronic technology that allows researchers to observe, record, and quantify feeding behaviors of arthropods whose mouthparts penetrate into opaque host tissues, and therefore cannot be directly visualized in real-time. This revolutionary technology was originally invented^18^ to study the feeding behaviors of very small plant sap-feeding insects on host plants. The technology has subsequently been improved several times^19–21^, but the basic principle remains the same. An AC (alternating current) or DC (direct current) signal is conveyed to the plant via a referent electrode in the soil^22^. The arthropod (tethered with a recording electrode of thin, solid-gold wire glued to its dorsum) is placed on the plant. When it inserts its mouthparts, current is conveyed to the instrument for signal processing and then to a computer. Changes in output voltage over time create electrical patterns (waveforms) displayed on the computer. Waveforms can be correlated with highly specific behaviors, such as mouthpart movements, direction of fluid flow, salivation, ingestion, puncturing of specific cells, and pathogen acquisition/inoculation^22^. Three generations of major EPG technology designs have occurred, in parallel with advancements in electronics^17,22^. The first-generation (AC) monitors^18,20^ (no longer manufactured); used high AC applied signal and fixed-low amplifier sensitivity (input resistor or Ri; 10^6^ Ω). The second-generation (DC) monitor^21^ (still marketed); uses low-to-high DC applied signal and fixed, high Ri (10^9^ Ω). More recently, a third-generation (AC–DC) monitor was introduced^19^ as a culmination of design and signal analysis comparisons among the previous designs. The AC–DC monitor has selectable AC or DC applied signal and selectable Ri (10^6^ to 10^10^ Ω plus 10^13^ Ω).

EPG has been widely used in studies of feeding behavior/physiology of plant-feeding, piercing-sucking hemipteroid insects^17,22^. EPG has made possible virtually all present knowledge of the role of feeding in vector-mediated transmission of plant pathogens^22^. In addition, EPG has been instrumental in gaining insights into and improvements in pest management^17^, such as: (i) targeting insecticide modes of action^23,24^; (ii) improving host plant resistance^25,26^; and, (iii) targets for RNAi^27^.

Application of EPG to the study of tick feeding behavior and tick-host interactions could be a profoundly enabling solution to investigate, in unprecedented detail and real-time resolution, the temporal intricacies of tick feeding behaviors and tick-host interactions. We hypothesize that EPG can be tailored to investigate on-host tick feeding and that ticks will exhibit a complex assembly of waveforms representative of specific behaviors while attached to the host. Therefore, the objectives of this study were to: (i) demonstrate that AC–DC EPG can successfully be used to monitor on-host tick feeding behaviors; (ii) determine whether ticks produce distinguishable EPG feeding waveforms; and, (iii) qualitatively and quantitatively describe/compare tick EPG feeding waveforms performed by two tick species during the early stages of slow-phase tick feeding^28^.

## Methods and materials

### Ticks and tick maintenance

Pathogen-free, adult female *Dermacentor variabilis* (American dog tick) and *Amblyomma americanum* (Lone star tick) (Ecto Services, Inc., Henderson, NC), ca. 12–14 weeks post-molt, were used in this study. Ticks were maintained in humidified chambers within environmental incubators at 98% RH, 26 °C, and with a 12:12 L:D cycle until use.

### Calf maintenance and tick infestation

All study activities involving animals were reviewed by the Kansas State University Institutional Animal Care and Use Committee and performed in accordance with the approved study-specific protocol and ARRIVE guidelines. Four Holstein or Holstein-Jersey steers between 4–8 months of age were used as tick-feeding hosts. Feeding patches for ticks were applied to calves by shaving an area on their back behind the shoulder and attaching a stockinet using a veterinary-approved adhesive. Ticks (~ 5 total per patch, 2–3 *D. variabilis* and 2–3 *A. americanum*) were placed together in the stockinet and allowed to attach to the calf. During tick infestation, calves were housed indoors and restrained in stanchions to prevent them from disturbing the tick-feeding patch. Calves were fed a complete grain diet at 2% bodyweight per day, hay, and water ad libitum.

Stanchioned calves had free vertical movement and received no pharmacological drugs to avoid reducing other natural movements; by comparison, electrophysiology studies normally require use of fully sedated animals, constraining recording durations. During the recording process, these awake calves regularly would stand/lay, perform normal movements, and other bodily functions within the confines of the stanchion. One head stage amplifier was mounted on the back of each calf near the wired tick; thus, the amplifier moved with the calf. Investigators observed real-time data capture of all waveforms. When a calf laid down, such large, jolting movement occasionally caused a voltage peak in the recordings (which was inserted as an observation in real-time on the recording), but the other normal calf behavior movements did not affect the recordings.

### Electropenetrography

Individual ticks were wired after attachment to the calf host. Ticks were tethered with a 38.1 µm thin, gold wire (sold as 0.0015 in., Sigmund Cohn Co., Mt. Vernon, NY) following the methods previously described^29^, using silver glue whose recipe is described therein. In brief, gold wire was glued to a copper wire soldered to a brass nail, then was bent into a small loop at its other end; the loop was then glued to the dorsum of the feeding tick. While multiple recordings were made, not all were considered high enough quality for measurement and publication. Recordings, of different time durations, were made early during slow-phase tick feeding, between 20 and 48 h post-infestation from a total of four ticks (two *D. variabilis* and two *A. americanum*). Three recordings were analyzed of *D. variabilis* ticks (tick 1 for 70.2 and 14.0 min, and tick 2 for 24.3 min); one recording was analyzed for each *A. americanum* ticks (tick 3 for 276.5 min and tick 4 for 172 min). We recorded one tick at a time per host (no simultaneous recordings of multiple ticks on the same host at the same time). The two *D. variabilis* were recorded on the same calf at different, non-overlapping times on the same day. The two *A. americanum* ticks were recorded on two separate calves for overlapping time periods over 2 days. All recordings made in this study occurred before any tick began to develop any noticeable abdominal expansion.

Recordings were made using a four-channel AC–DC electropenetrograph manufactured by EPG Technologies, Inc. (Gainesville, FL, andygator3@gmail.com). The instrument is similar to that introduced in^19^, described further in^22^, with block diagram in^30^. Head stage amplifiers and applied signal electrodes were secured on the calf near the tick attachment location using a combination of veterinary-approved adhesive and adhesive tape. Applied voltage was 350 mV AC and amplifier sensitivity (input resistor or Ri level) was set to 10^8^ Ohms for most recordings. Analog signals were acquired and digitized via a DI-710 board and Windaq Lite software (both from DATAQ Instruments, Akron, OH) using sample rate of 100 Hz. Waveforms were re-played for measurement using Windaq Waveform Browser (DATAQ). Pre- and post-rectification signals were simultaneously recorded and checked to ensure that they were identical; if not, the offset function was used to remove rectifier fold-over of the output signal and retain native polarity (positive- or negative-going property) of the waveform^31^. Instrument gain for all the recordings was 2000X. As needed, a piece of a grounded thermal mylar blanket was draped over the assembly of head-stage amplifier and feeding tick to reduce ambient electrical noise during recordings.

EPG waveform terminology used was similar to the system previously published^29^. Briefly, a ‘waveform’ is a series of output voltage changes over time that, together, form a pattern discernible to the human eye. When describing further structural composition of waveforms, a hierarchical system (from largest to smallest) of waveform ‘family’ and waveform ‘type’ is used. A waveform family is a recognizable waveform appearance visible to the human eye at a coarse level, or compression (on the X-axis) level of 1–2 min per vertical division in the screen view. A family can be a single, undividable pattern, or have obvious components that can be further subdivided as waveform ‘types’ when compression is spread out to seconds per division. Thus, in the present study, some waveforms were divided only to the family level, while others were further divided to the type level. Within a family, types can be single, that is, never repeated in a measured event (see below for definition of ‘event’), or types can be highly repetitive, with several types grouped together to form ‘episodes’ that are consistently repeated within an event of the family.

A waveform ‘event’ is not part of the above hierarchical naming convention, but an independent identification that is important for quantitative measurement of waveform durations, counts, and frequencies. An event consists of a single, uninterrupted occurrence of a waveform family or type, whose duration is measured for quantification. No matter what aspect of a waveform in an event is measured (duration, frequency), each individual measurement ultimately becomes a single observation in a dataset for statistical analysis. Each event has a sequence-specific location in the recording; thus, each event is temporally unique. A researcher can choose to name an event at the family level [e.g., all of Dv1 until interrupted by Dv2 (see below)] or at the type level [e.g., Dv1a separate from Dv1b, and so on (see below)]. Certain families (e.g., Dv3, see below) are considered events at the family level, with no further division into types. Others can be considered events at either the family level (e.g., Dv1 for durations herein) or the type level (e.g., Dv1a for frequencies herein).

Durations and counts of events at the waveform family level were summarized and descriptive statistics were calculated using the Ebert v. 1.0 and Backus v. 2.0 SAS (v. 9.4) analysis programs (SAS, Cary, North Carolina, U.S.A.) (downloadable from https://crec.ifas.ufl.edu/extension/epg/epg_workshop.shtml), generating means and standard errors for the following, standardized variables for each species: (1) Waveform Duration per Event per Insect (WDEI), (2) Number of Waveform Events per Insect (NWEI), and (3) Waveform Duration per Insect (WDI)^31,32^. (The above, standardized variable names were used to be consistent with other EPG studies, even though ticks are not insects.) In addition, number of episodes (see definition below) of Family 1 waveforms were manually counted within selected, naturally terminated events, i.e., Family 1 events that both preceded and followed recorded Family 2 events (see below). All such naturally-terminated events were counted for *D. variabilis*, while 20 randomly-selected Family 1 events per tick were counted for the longer *A. americanum* recordings that contained many Family 1 events. Manual episode counts were summarized for each species for the following variables: (1) mean (naturally terminated) event duration, (2) mean number of episodes per event, (3) mean number of episodes per sec. While sample sizes of recordings per species were considered sufficient for characterization and descriptive statistics, they were not sufficient for statistical testing of the above duration-based variables or between species.

In contrast, in-depth measurements of waveform frequency (i.e., number of peaks per sec, or Hz) were statistically comparable among waveform types in Families 1 and 2, for individual ticks within species. This was done because frequencies (or frequency-amplitude combinations) have been shown in other EPG studies to be highly characteristic of different waveform families and types. For Family 1 types, frequencies were measured for 10 representative episodes per tick. For Family 2 types, frequencies were measured for all events in each recording (See “Results”). The same SAS programs cited above were again used to generate means and standard errors, but also to perform mixed model Analysis of Variance to statistically compare among untransformed means by and among ticks and waveforms^31,32^. Means were considered significantly different at α = 0.05.

## Results

### Overview

To demonstrate the application of EPG to study tick feeding behavior, we characterized and compared the feeding of two tick species (*D. variabilis* and *A. americanum*) during the early stages of slow-phase tick feeding (~ 20–48 h post-infestation). Baseline waveform levels for an unattached tick standing on calf were determined at the end of recording by forcing the tick to detach from the calf. Both on-calf and off-calf baseline voltage levels were far below the voltage level for all tick feeding waveforms observed. Therefore, all waveforms were monophasic positive (i.e., positive-going) in polarity^30^. Three families of waveforms, based strictly on appearance, were characterized.

Waveforms were hierarchically categorized into three families and seven types within families, named consistently for both species. Type categorization was supported by the statistical frequency analysis, whose results are described within each section below. Waveforms were considered very high-frequency (very fast) at 10.0–25 Hz., high-frequency (fast) at 6.0–9.9 Hz., medium-frequency at 4.0–5.9 Hz. (medium), low-frequency (slow) at 2.0–3.9 Hz., very low-frequency (very slow) at 0.1–1.9 Hz., or flat at 0.0 Hz. Frequencies for each waveform type within each tick were highly consistent. Frequencies were significantly different among different waveform types (*P* < 0.0001 within each tick species) but, unless otherwise stated below, were not significantly different between ticks within a species.

### Family 1

The most common waveform family, Family 1, occurred in the background throughout all recordings with virtually no cessation except interruption by the other two families. Family 1 waveforms were similar between ticks within each species but were interestingly different between species. Therefore, we chose specific Family names for each species: Dv1 (for *Dermacentor variabilis* Family 1) and Aa1 (for *Amblyomma americanum* Family 1).

**Figure 1:**
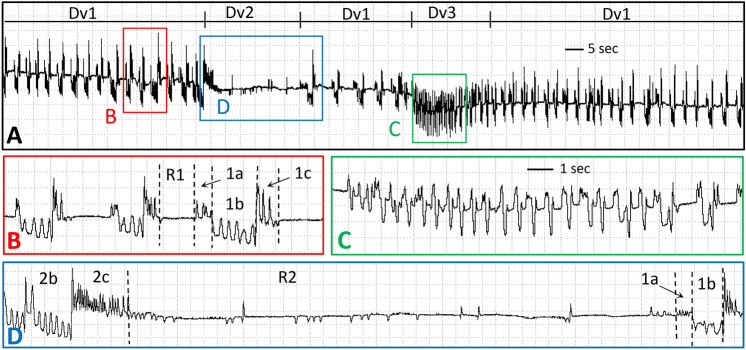
EPG waveforms for *D. variabilis* feeding approx. 36 h after attachment to calf. (**A)** Compressed view of three Dv1 events and intervening Dv2 and Dv3 events. Labelled boxes contain waveform segments that are expanded in parts with the same letter label. Windaq gain 64×. (**B)** Two and a half episodes of Dv1 showing all four types, Dv1a, Dv1b, Dv1c and DvR1, but with Dv removed from each label to save space. (**C)** One event of Dv3. (**D)** One event of Dv2, plus the start of the next Dv1 event. See narrative for further descriptions of waveform names. Scale bar in part C and Windaq gain of 128 × same for parts B – D.

**Figure 2:**
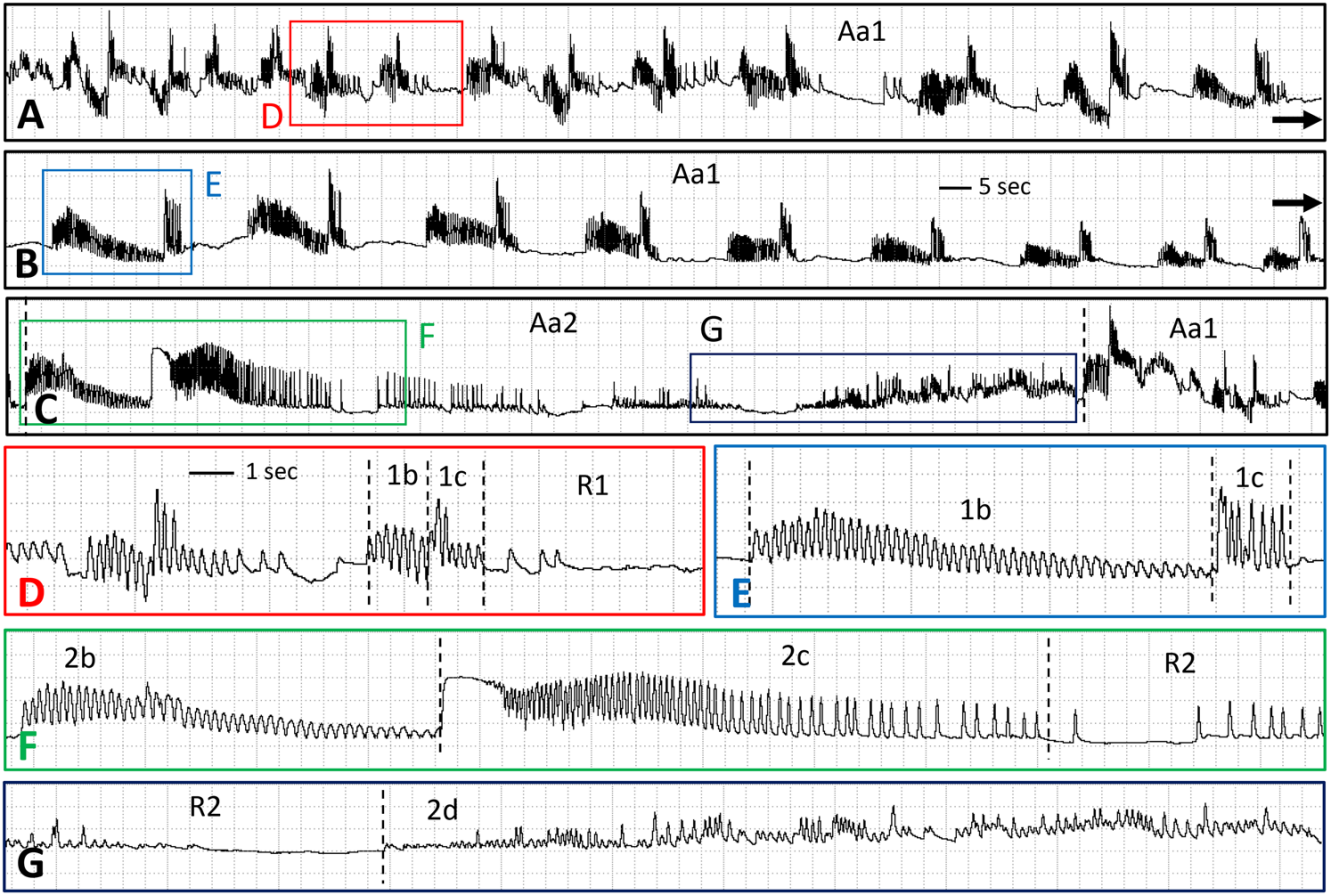
EPG waveforms for *A. americanum* feeding approx. 36 h after attachment to calf. (**A–C)** Compressed view of one event of Aa1, showing appearances of successive episodes during the progression of the event through three rows of waveforms. Labelled boxes contain waveform segments that are expanded in parts with the same letter label. Scale bar in part **(B)** same for parts **(A,C)**. Windaq gain 64×. (**D)** Two episodes of Aa1 showing four out of four types, Aa1b, Aa1c and AaR1, but with Aa removed from each label to save space. (**E)** A later episode of Aa1 showing lengthened Aa1b. (**F)** First third of one event of Aa2, showing three out of five types, Aa2b, Aa2c, and the first part of AaR2. (**G)** Last third of one event of Aa2, showing last part of AaR2 and all of Aa2d. See narrative for further descriptions of waveform names. Scale bar in part D and Windaq gain of 128 × same for parts **(D–G)**.

Family 1 was composed of highly stereotypical and repeating episodes, rapidly recurring about every 2.5 s for dozens of episodes per event of Dv1 for *D. variabilis* tick 1 (Fig. 1A), but more slowly (every 10 s) for tick 2 (Table 1). Dv1 events lasted 3–5 min each (Table 2). In contrast, both *A. americanum* ticks (3 and 4) performed Aa1 similarly. Each Aa1 episode was much longer and less frequent (about once every 20 s) than for Dv1, with fewer than 20 episodes in each 3–6 min event for many measured events of Aa1 (Fig. 2A, Table 1).

**Table 1:** Descriptive statistics for early, slow-phase feeding episodes of adult, female *D. variabilis* and *A. americanum* Family 1 waveforms measured within naturally terminated events.

Tick no.	Recording no.	Recording duration (min)	Number of episodes counted in selected Family 1 events (all naturally terminated)			
			N	Mean event duration (s)	Mean no. episodes per event	Frequency (mean episodes per s)
***D. variabilis***						
1	1 & 2	84.2	9	314.2 ± 55.7	125.6 ± 34.7	0.40
2	3	24.3	1	233.4	24.0	0.10
***A. americanum***						
3	4	276.5	20	185.9 ± 20.2	11.3 ± 1.7	0.06
4	5	172.0	20	370.6 ± 66.9	17.3 ± 2.6	0.05

Mean ± standard errors.

**Table 2:** Waveform duration per event per insect (WDEI) means ± standard errors (in sec) during early, slow-phase feeding of adult, female *D. variabilis* and *A. americanum*.

	N	WDEI (s)		N	WDEI (s)
*D. variabilis*			*A. americanum*		
Waveform Dv1	17	309.0 ± 94.5	Waveform Aa1	74	849.8 ± 627.8
Waveform Dv2	15	24.3 ± 7.8	Waveform Aa2	73	126.8 ± 1.4
Waveform Dv3	1	19.9			

*N* number of events.

Family 1 episodes were divided into four subtypes, termed 1a, 1b, 1c and R1 (Fig. 1B), although Type 1a (thus, Dv1a) was only performed by tick 1. It is possible that Dv1a was not seen in the tick 2 recording because that recording was slightly noisier than the recording of tick 1; alternatively, the behavior represented by Type 1a may not be conserved, as described in “Discussion”. Type Dv1a comprised a high-frequency series of peaks (Table 3) all of the same short amplitude (Fig. 1B). Type 1a was not performed by *A. americanum* in our recordings.

**Table 3:** Results of frequency analysis of tick waveform types.

	Type 1a		Type 1b		Type 1c		Type R1	
	N	Hz	N	Hz	N	Hz	N	Hz
*D. variabilis*	10	8.45 ± 0.53 a	20	4.30 ± 0.35 b	20	8.07 ± 0.23 a	20	0.00 ± 0.00
*A. americanum*	0		20	4.44 ± 0.11 ab	20	6.02 ± 0.32 a	20	0.49 ± 0.17 c

	Type 2a		Type 2b		Type 2c		Type R2	
	N	Hz	N	Hz	N	Hz	N	Hz
*D. variabilis*	5	8.41 ± 0.76 a	8	4.19 ± 0.35 b	8	9.26 ± 0.54 a	8	1.6 ± 0.19 c
*A. americanum*	6	4.83 ± 0.58 ab	23	3.74 ± 0.07 b	23	5.47 ± 0.11 ab	23	0.38 ± 0.08 d

	Type 2d							
	N	Hz						
*D. variabilis*	0							
*A. americanum*	18	1.33 ± 0.35 c						

Mean ± standard errors in Hz.

Same lower case letter indicates values were not significantly different.

Type 1b immediately followed Dv1a or was the first component of a Family 1 episode in *A. americanum* (Aa1b). Type 1b consisted of medium-frequency, short-to-medium-amplitude peaklets that occurred on a different voltage level, either lower (Dv1b; Fig. 1B) or slightly higher (Aa1b; Fig. 2D) than the voltage level preceding it. Type 1b durations were short in *D. variabilis* as well as in early episodes of *A. americanum* (Table 2). However, Aa1b sections in each episode gradually lengthened in duration over time, also becoming shorter in amplitude and eventually tapering off (compare Fig. 2D,E). Mean frequencies were significantly different between ticks within species. Dv1b of ticks 1 and 2 were numerically and significantly different (2.83 ± 0.36 for tick 1; 5.78 ± 0.10 for tick 2) (df = 1, 18, F = 63.18; *P* < 0.0001). Aa1b for ticks 3 and 4 appeared less numerically different, yet also significantly different between ticks (4.79 ± 0.10 for tick 3; 4.10 ± 0.11 for tick 4) (df = 1, 18, F = 21.01*P* = 0.0002). There was little variation in frequency of Aa1b among episodes within each tick.

For both species, Type 1c was a high-frequency (Table 3) series of peaks, short in duration (Figs. 1B, 2E), but higher in amplitude than the preceding Type 1b. Frequencies of 1c were not significantly different between ticks within species.

The fourth component of a Family 1 episode consisted of short or variable durations of flat or near-flat waveform. Because this waveform was located at a voltage level above baseline, this likely represents a period of no or reduced behavioral activity while the tick was still attached to the calf. Thus, the waveform was termed Resting (R), Type 1 (R1), for Family 1 (Figs. 1B, 2D). In addition, frequencies for *A. americanum* ticks 3 and 4, while very low, were nonetheless significantly different (0.91 ± 0.28 for tick 3; 0.08 ± 0.03 for tick 4) (df = 1, 18, F = 8.9; *P* = 0.008). Thus, while no peaks occurred during DvR1, a few stray peaks occurred occasionally during AaR1 (compare Figs. 1B and 2D).

Family 1 episodes for *D. variabilis* were uniformly short in duration and rapidly repeated (Fig. 1) before abruptly transitioning into Family 2. In contrast, Family 1 episodes for *A. americanum* gradually lengthened in duration as the episodes were repeated over time between Family 2 events (Fig. 2). This lengthening of Aa1 episodes over time occurred primarily because of the lengthening of Aa1b, described above. Also, R1 resting intervals gradually lengthened between Aa1 episodes, until they reached maximum duration shortly before the recording abruptly transitioned to Family 2. Constant performance of Family 1 episodes was the background tick feeding behavior in our recordings, on which were superimposed two more waveform families.

### Family 2

On a predictable and regular cycle for each recorded tick, a distinctive waveform change occurred, comprising Family 2. This change occurred about every 5 min for both *D. variabilis* ticks, but every 3 or 6 min for *A. americanum* ticks 3 and 4, respectively (Table 2). Despite their regularity, due to varying recording durations for each tick, varying numbers of Family 2 events were measured, i.e., 6 events (tick 1), 2 (tick 2), 10 (tick 3), and 13 (tick 4). Family 2 was composed of five types.

Type 2a, like Type 1a, was not recorded for every episode of Family 2, i.e., 62.5% of episodes (both ticks 1 and 2) had 2a for *D. variabilis*, but only 26% of episodes (only tick 4) for *A. americanum* (Table 3). Because of its relative rarity in *A. americanum* recordings, Type 2a is not shown in Fig. 2. However, its appearance was very similar to Type 1a (for *D. variabilis*, Fig. 1B), with statistically similar high frequency and short duration.

Type 2b was nearly identical in appearance to Type 1b for both tick species (compare Figs. 1B,D, 2D–F), except that Aa2b always looked like the longest-duration version of Aa1b. Like 1b, both species performed medium-frequency peaks. Mean frequencies of Dv2b were significantly different between ticks 1 and 2 (3.72 ± 0.24 for tick 1; 5.58 ± 0.04 for tick 2) (df = 1, 6, F = 17.83; *P* < 0.0001) but not significantly different for Aa2b between ticks 3 and 4 (3.66 ± 0.13 for tick 3; 3.79 ± 0.09 for tick 4).

Type 2c was always performed in both tick species, but its appearance varied by species. In *D. variabilis* recordings, Dv2c was similar to Dv1c; a very high-frequency peak burst on a higher voltage level, distinctly separate from Type 2b and relatively short in duration (Fig. 1D). In *A. americanum*, however, Aa2c was different in appearance from Aa1c. Each Aa2c started with an abrupt jump in voltage level from the preceding, low-amplitude end of Aa2b. This short, flat, then usually declining-voltage-level section of Aa2c abruptly transitioned into a much longer-duration section distinctly resembling a comb with ever-widening tine-like peaks (Fig. 2F). The comb started at a very high frequency of 20–25 Hz, then gradually slowed through medium to low frequency of 3–5 Hz, eventually tapering off to flat or near-flat (R2, below). When calculated over a full duration of Aa1c, mean frequency was medium (Table 3).

Following Type 2c, another resting section (near-flat R2) occurred. Type R2 was different in appearance for *D. variabilis* versus *A. americanum*. DvR2 was quite long in duration and included frequent (at the beginning), short dips downward and occasional short, upward peaks (Fig. 1D). After a few sec, the downward dips ended, and the short peaks sometimes became more frequent (Fig. 1D); however, usually there were no peaks at the end of DvR2. DvR2 ended abruptly with the start of new Dv1 episodes, but with longer DvR1 resting periods (Fig. 1A). After four to six of such special Dv1 episodes, a gradual decline in voltage level sometimes led into Dv3 (see below).

AaR2 was different from DvR2. AaR2 began with a distinct lengthening of the flat line between Aa2c peaks, followed by a short section almost as flat as R1 but with slight undulations that gradually became spikier until a series of very short, high-frequency spikes erupted, gradually increasing in amplitude but still very irregular in frequency (Fig. 2G). After a fairly long duration of flat waveform interspersed with short peak bursts, AaR2 abruptly transitioned to Aa2d (see below).

Unsurprisingly given the above differences in R2 waveform appearances, especially varying numbers and arrangements of peaks, R2 frequencies appeared to be different between ticks in each species. *D. variabilis* frequencies were very low to low and significantly different between ticks 1 and 2 (1.37 ± 0.15 for tick 1; 2.31 ± 0.17 for tick 2) (df = 1, 6, F = 11.26; *P* = 0.0153). Similarly, *A. americanum* frequencies were very low and also different between ticks 3 and 4 (0.64 ± 0.15 for tick 3; 0.19 ± 0.05 for tick 4) (df = 1, 21, F = 9.86; *P* = 0.0049).

Uniquely for *A. americanum* (Table 3), 78% of Aa2 episodes had a variably long duration of irregular-frequency and -amplitude peaks at the end of each event (Fig. 2G) termed Type 2d (thus, Aa2d). Frequencies were significantly different between the 9 episodes for each tick (2.04 ± 0.61 for tick 3; 0.62 ± 0.12 for tick 4) (df = 1, 16, F = 5.21; *P* = 0.0364). Aa2d ended with an abrupt transition to Aa1a, the start of a new cycle of Family 1 episodes.

### Family 3

Due to shorter recording durations, only one Dv3 event was found for *D. variabilis* (Fig. 1D). Family 3 was strikingly different in appearance from the two previous families, and less uniform in appearance because its appearance evolved over time; thus, no types were assigned. Dv3 started with a Dv1b-like series of broad peaks that gradually increased in amplitude, declined in voltage level, and developed into two-peaked, M-shaped plateau-like structures that were repeated several times (Fig. 1C). These two-horned plateaus gradually evolved into Dv1a and Dv1b, eventually resuming the background behavior of repetitive Dv1. Despite longer recordings for *A. americanum* than for *D. variabilis*, no Family 3 events were observed for *A. americanum*.

## Discussion

A major challenge to the development and rigorous evaluation of host-level tick and TBP intervention strategies is the lack of transparency of temporal events and behaviors that occur at the tick attachment site—the interface of tick-host-pathogen interactions. The occurrence of these behaviors within host skin obstructs ready investigation of ‘normal’ tick-host interactions and impedes evaluation of how chemical control measures or pathogens specifically alter these interactions to inhibit successful tick feeding or facilitate transmission, respectively. Adapting AC–DC EPG to study the intricate behaviors of blood-feeding arthropods on-host can potentially transform blood-feeding arthropod research.

Electronic instruments have previously been used to record the feeding behavior of blood-feeding arthropods (~ 20 publications), including with argasid ticks^33,34^. Recent instrument designs use high-sensitivity electromyography (EMG) amplifiers, like those used by electrophysiologists. EPG and EMG are easily confused, but there are distinct differences between them, namely, the placement of the referent electrode and amplifier design. The electrical circuit for EPG includes both the arthropod and its host. The recording electrode is attached to the arthropod while the referent electrode is attached to the host. Waveforms are derived from electrical currents carried by ionized fluids in the mouthpart canals and foregut of the arthropod. The EPG monitor detects a mixture of (i) resistance to/conductance of current flow (R component); and, (ii) non-neural biopotentials, i.e., streaming potentials generated during fluid flow (emf component). In EMG, the electrical circuit includes only the arthropod; both the recording and referent electrodes are attached to different parts of the arthropod body. EMG instruments are designed to record strictly biopotentials, i.e., muscle/action potentials nearest the recording electrode; in EPG parlance, they record only emf component from neural sources. EMG instruments necessitate higher Ri levels, extensive band pass filtering, and differential amplification to detect these tiny biopotentials, compared with EPG instruments. More information on EPG/EMG electronics is here^17,22,35^.

Thus, EMG instruments previously used for tick recordings were designed to detect biopotentials originating in muscles^35^ controlling ingestion^35^, not salivation. Far more information is available if recording amplifiers are also sensitive to electrical resistance/conductivity of fluid (e.g. saliva, blood) flow. These include: (i) electrical resistance from opening/closing of valves/pumps in the foregut as well as mouthpart movements/depth; and, (ii) electrical conductivity related to biochemical composition of saliva^20,22^. While a few EMG papers have identified trace salivation and mouthpart movement biopotentials^33,34^, their electrical resistance information is likely incomplete. In addition, a major benefit of AC–DC EPG is the far wider range of selectable amplifier sensitivities, to detect all signal types from resistance-only to biopotential-only and mixtures of both^19,22^. Another major benefit of AC–DC EPG is that the head stage amplifier is relatively sturdy, and can be attached to a gently-moving, unsedated host animal (as in our study), allowing for increased recording durations, especially important for studying ixodid tick feeding behaviors. Previous EMG studies used sedated mice because the amplifier was too delicate to tolerate host movement. Thus, AC–DC EPG will likely provide a far wider and more operationally useful view of ixodid tick behaviors and physiologies than EMG.

In our study, we used AC–DC EPG to record and begin to characterize on-host feeding waveforms (behaviors) for two ixodid tick species of medical and veterinary significance during periods early in slow-phase tick feeding (~ 20–48 h post-infestation). We demonstrated that: i) AC–DC EPG can be used to monitor tick feeding behaviors on an awake animal host; ii) ticks produce a constant series of differentiable and definable waveforms while feeding on-host; and, (iii) a similar series of recognizable waveforms were produced by two ixodid tick species at a comparable feeding stage.

Another challenge to studying ixodid tick feeding behaviors is the extreme duration adult ticks feed, normally 6–9 days. This study provides brief ‘snapshots’ of adult tick feeding behaviors within the early stages of slow-phase feeding; a timeframe during which chemical control measures are expected to work and when pathogen transmission often occurs. During slow-phase feeding, adult ixodid ticks inject a temporally orchestrated cornucopia of salivary proteins^10^ to complete attachment, evade host immune responses, prepare the feeding lesion, and begin feeding/ingesting, all processes masked within host tissue. Previous studies of mechanisms underlying tick-feeding success have primarily used various combinations of morphology, histology, transcriptome, and proteome analyses at defined time points post-infestation or attachment. The latter often encompasses a variable window of time rather than a specific time^10,15,28,36–38^. Previous studies examining specific tick tissue functions are rarer, and commonly require: (i) interruption of tick feeding, with or without dissection of the tissue target; (ii) adaptation of ticks to artificial feeding systems; (iii) use of sedated hosts (argasid tick studies); or, (iv) significant host discomfort^33,34,39–41^. Studies on real-time expression of precise on-host ixodid feeding behaviors are lacking, largely due to absence of tools to investigate them. Addressing this deficit, we demonstrate that AC–DC EPG can be adapted to investigate on-host ixodid tick feeding behaviors in real-time, following a specific, behavior-triggered zero time point.

While EPG waveforms were similar enough between *D. variabilis* and *A. americanum* to be generally categorized into the same waveform families and types, there were interesting differences between species. Generally, *D. variabilis* performed waveforms faster than *A. americanum*. Episode repetition rate of the constantly-cycling background waveform, Family 1, was faster (every 2.5 s) for *D. variabilis* than for *A. americanum* (every 10 s). Similarly, although waveform types usually had stereotypical frequencies (peaks per sec), those of *D. variabilis* were commonly higher/faster than those of *A. americanum*.

In addition, certain waveform types were always performed by both species (tentatively termed conserved), while other types were rarely performed, either by one or both species (tentatively termed variable). For example, in waveform Family 1, Types 1b, 1c, and R1 were highly conserved, performed dozens to hundreds of times by all ticks in both species at stereotypical frequencies. In contrast, Type 1a was only performed by *D. variabilis* tick 1 and no other. It is possible that quality of recordings eliminated 1a in *D. variabilis* tick 2. However, because recordings for both the *A. americanum* ticks (3 and 4) were very high-quality, noise cannot be the sole reason for the loss of 1a in those ticks.

Similarly, for Family 2 waveforms, Type 2a was recorded in three out of the four ticks, but *A. americanum* tick 3 did not perform 2a, and certain events lacked 2a in the other three ticks’ recordings. Interestingly, Type 2d was exclusively observed in *A. americanum*. In contrast, Types 2b, 2c, and R2 were performed by all ticks of both species, in every Family 2 event recorded. Also, like their Family 1 counterparts, the 2b, 2c, and R2 frequencies were highly stereotypical for both species, again being medium-, high-, and low-frequencies, respectively. Thus, 2b, 2c, and R2 represent highly conserved behaviors; Types 2a and 2d represent variable behaviors.

Due to the repetitive, stereotypical and cycling background behavior of Family 1 waveforms for both tick species, we hypothesize that these frequent and repetitive waveforms are associated with salivation and specific mouthpart, pump or valve movements. Consistent salivation at this stage of the tick feeding process is likely required to prevent rejection from the host immune responses and prepare the feeding lesion^10,42–44^. We also hypothesize that different salivary secretions (perhaps different chemistries from different acini cell types) flow from the mouthparts during conserved 1a, 1b, and 2c. Perhaps the variable waveform 1a represents a less-commonly secreted type of saliva. During R1 of Family 1, the ticks rest for a few seconds before beginning the salivation cycle anew.

During a second type of regularly cycling (but less frequent) behaviors, Family 2 waveforms are interspersed among Family 1 waveforms. It is possible that the Family 2 waveforms represent expulsion of a different salivary composition because the waveforms resemble Family 1 waveforms, but are longer in duration and more detailed in fine structure. Alternatively, the regularly cycling Family 2 waveforms may represent brief periods of fluid uptake and ingestion, which may occur less frequently during the first 48 h of ixodid tick feeding, enabling the tick to gauge its feeding progress or help replenish depleted resources. Again, the conserved performance of 2b and 2c suggest that they represent essential behavioral events, while the variable Types 2a and 2d represent less important or less frequently required behavioral events.

Neural control of tick salivary glands, including: (i) salivary gland innervation patterns; (ii) roles of associated neurotransmitters; and, (iii) temporal production of transient secretory vesicles, are all areas of interest^40^. EPG can be used to better understand these mechanisms underpinning salivary gland control, to study their ultimate functional effect. For comparison, different salivary fractions of hemipteran piercing-sucking insects can be secreted by each section of the principle salivary glands under voluntary control by the insect^45–48^. Future work to differentiate conserved versus variable waveforms could help in deciphering the biological meanings of these tick-feeding waveforms.

Our study is an introductory, proof-of-principle benchmark for future tick EPG research. We demonstrate the active and intricate behaviors performed by ixodid ticks, even for only a brief period of the extensive tick feeding process. Additional studies, with longer recordings and larger sample size, will be required to confirm whether Family 1 and 2 waveforms initially characterized as variable are truly idiosyncratic for the studied tick life stage and species. Future studies with longer recordings are also needed to fully qualitatively and quantitatively characterize the temporal sequence of on-host tick feeding behaviors. Studies deciphering the behavioral and functional activities associated with individual waveform families and types will be required to interpret their biological significance.

Despite the medical and economic importance of ticks and other blood-feeding arthropods, effective control measures are limited. The imperative need for development of new mitigation strategies requires novel means of closing persistent knowledge gaps about arthropod blood feeding. AC–DC EPG provides a platform to delineate intricate and temporal feeding behaviors of ticks and other blood-feeding arthropods^49^. Such fundamental studies are required to understand how chemical control interventions, pathogens, or host immunity functionally alter feeding behaviors. Once such benchmark studies are completed, EPG can be used to rigorously evaluate how management methods, pathogens, etc. alter blood-feeding arthropod feeding behavior(s) using real-time quantitative measurements. Further, EPG can be used to identify perfect zero time points based on initiation of a specific behavior rather than using time post-infestation/attachment. For ixodid ticks, successfully capturing a true zero timepoint has been consistently difficult; achieving this task will have profound implications for interpretation of experimental results. Accordingly, we contend that AC–DC EPG is a uniquely enabling technology, with transformative potential to widely open previously unrealizable investigative possibilities to study blood-feeding vector-host–pathogen interactions.

## Data Availability

All data generated or analyzed during this study are included in this published article.
